# Atypical Reversible Leucoencephalopathy Syndrome after Bevacizumab/Folfox Regimen for Metastatic Colon Cancer

**DOI:** 10.1155/2014/391256

**Published:** 2014-10-21

**Authors:** Narimane Salmi, Ibrahim Elghissassi, Khadija Bellahammou, Asmaa Lakhdissi, Hind Mrabti, Hassan Errihani

**Affiliations:** ^1^Department of Medical Oncology, National Institute of Oncology, Rabat 10000, Morocco; ^2^Université Mohammed V-Souissi, Rabat 8007, Morocco

## Abstract

We are reporting a case of multifocal reversible leucoencephalopathy syndrome induced by chemotherapy based on Folfox-Bevacizumab regimen. A 44-year-old female, with no history of hypertension, received a chemotherapy based on Folfox-Bevacizumab for her metastatic colon cancer (5 FU: 325 mg/m^2^ d1 by intravenous infusion, Oxaliplatin 80 mg/m^2^ d1, and Bevacizumab: 7.5 mg/Kg d1). During the fourth cure, she presented delirium, seizures, and visual disturbances. The computed tomography (CT) of the brain showed hypodense lesions of the white matter of frontal, parietal, and occipital lobes, which were bilateral and symmetrical. The clinical table was reversible under symptomatic treatment.

## 1. Introduction

Leucoencephalopathy postchemotherapy is a very rare complication that affects the central nervous system. Posterior reversible leucoencephalopathy syndrome (PRLS) is the most described in the literature [1]; it is characterized by an impairment of the white matter of the posterior cerebral hemispheres. In some cases, this syndrome can affect the anterior hemispheres, gray matter, brainstem, and basal ganglia [2–4].

We describe an unusual case of diffuse leucoencephalopathy syndrome after chemotherapy based on Folfox-Bevacizumab (5 FU: 325 mg/m^2^ d1 by intravenous infusion, Oxaliplatin 80 mg/m^2^ d1, and Bevacizumab: 7.5 mg/Kg d1) affecting the central nervous system.

## 2. Case Presentation

We are presenting a case of a 44-year-old female, without any particular medical history. She is treated for a metastatic colon cancer for which she received chemotherapy based on Folfox-Avastin (5 FU: 325 mg/m^2^ d1 by intravenous infusion, Oxaliplatin 80 mg/m^2^ d1, and Bevacizumab: 7.5 mg/Kg d1). She has received a total of four cures. The blood pressure and the proteinuria were controlled at each cure and they were normal. The first three cures were well tolerated except a grade II diarrhea. During the fourth cure, the patient has developed laryngeal spasm during the infusion of Oxaliplatin; a day after chemotherapy, she has presented with a deviation and hypoesthesia of the tongue associated with swallowing disorders; the blood pressure was 17/9 mmhg.

Due to this clinical figure, we performed a computed tomography (CT) of the brain which was normal. Magnetic resonance imaging (MRI) of brain was not done because it was not available in our institute. The cerebrospinal fluid from lumbar puncture was clear and colorless, and the cytological examination did not find cancerous cells.

Five days later, our patient developed severe symptoms including dysarthria, tonic-clonic seizures, visual disturbances, headache, confusion, delusions, agitation, and behavioral disorders, with a sensory deficit in the lower limbs and distal motor weakness in the left upper limb. The blood pressure was 16/11 mm Hg, heart rate was 115 beats per minute, arterial oxygen saturation was 98%, and body temperature was normal without any signs of infection or sepsis. The complete blood count showed a thrombocytopenia at 49000 with normal hemoglobin and leucocytes.

**Figure 1:**
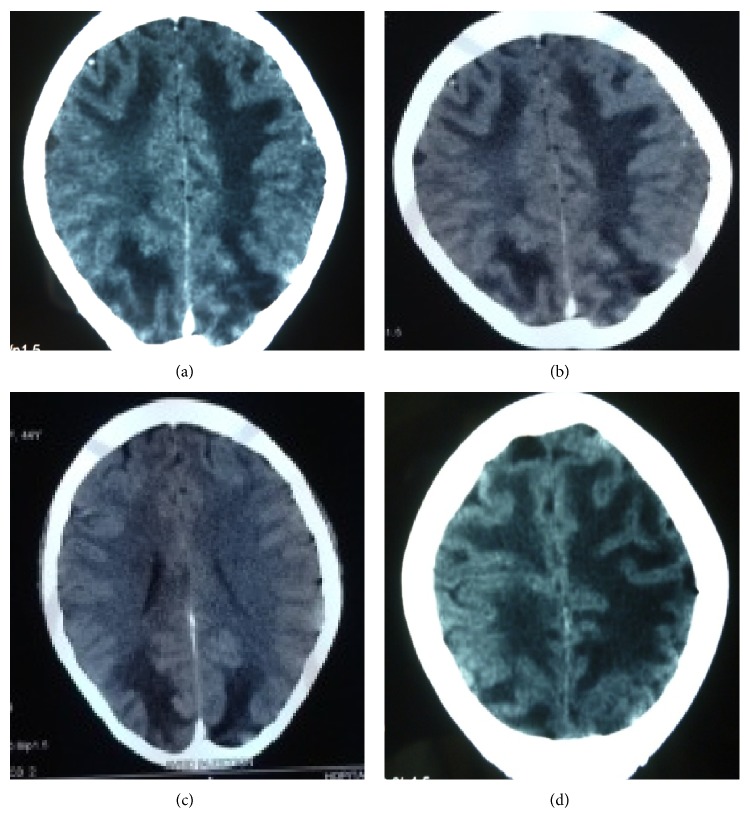
Brain CT scan with contrast shows subcortical hypodensities in the frontoparietooccipital region of white mater bilaterally and symmetrically without contrast enhancement.

Given this clinical figure, another brain computed tomography was performed in the fifth day resulting in hypodense, symmetrical, and bilateral lesions of the white matter of the frontal, parietal, and occipital area (Figure 1). The patient was treated with intravenous nicardipine, 1 mg every 15 minutes until normalization of the blood pressure and oral phenobarbital 150 mg a bid. After 48 hours of monitoring, we found that the blood pressure was back to normal (12/7 cmHg); then the antihypertensive therapy was not necessary. We did not give her a steroid therapy and we maintain oral phenobarbital.

Few days under symptomatic treatment, neurological examination showed a gradual return to normal and complete resolution of the neurological deficit. The diagnosis of postchemotherapy leucoencephalopathy was retained. The chemotherapy was stopped and the patient died two months later due to the progression of her disease.

## 3. Discussion

Posterior reversible leucoencephalopathy syndrome was first introduced in 1965 by Hinchey et al. [1]. In fact, it is probably a misnomer; this syndrome is not always reversible and is not necessarily limited to the posterior regions of the brain.

The pathophysiology of this syndrome remains debatable; it involves a diffusion of plasmatic proteins into the extracellular space. Two hypotheses have been advanced: the vasogenic and cytotoxic theories.

In the first hypothesis, the increase in blood pressure exceeding the cerebral autoregulation leads to a vasodilatation and a formation of vasogenic edema [1, 5, 6].

In the second hypothesis, it suggests that a brutal and significant increase in blood pressure produces a cerebral vasoconstriction with an ischemia which causes an injury of endothelial cell and the formation of cytotoxic edema [5, 7].

Headache is the most sign found in the clinical figures [5, 7–10]; seizures have been reported in most series [1, 5, 8, 10, 11]; they are often resolutive after management of PRLS and after discontinuation of the responsible agent. However in some pediatric series the evolution has been done to the status of epilepsy disease [12, 13]. Altered mental status as lethargy, confusion, and disorientation in time and space has also been reported frequently in the literature [8, 9].

The clinical figure includes also blindness which is cortical type [4, 6, 7], nystagmus [2, 5], and cases of vomiting and nausea [8, 9]. Focusing signs have also been described [5]. Hypertension is a common sign [1, 5, 7, 8, 10, 14] but that is not always present [8, 14].

The magnetic resonance imaging (MRI) is the main test for diagnosis. It shows abnormalities signs affecting the white matter as hyperintense lesions on T2 and Flair and hypointense on T1 [1–3, 15].

In our case, MRI was not performed because our institute does not have an MRI unit, which probably delayed the diagnosis of leucoencephalopathy postchemotherapy in our patient.

This syndrome affects typically the posterior lobes (parietooccipital) [1]; that is why it is called “posterior reversible leucoencephalopathy syndrome.”

In its atypical form it is also responsible for a breach of the frontal lobes (28–82%), basal ganglia (42–45%), thalamus (28–68%), brainstem (28%), and subcortical substance (5%) [2–4].

The brain CT scan may be negative in early symptoms; it might be done after the persistence of symptoms which will lead to the diagnosis by showing abnormal signs in the white matter [16]. In our patient, the brain CT scan performed one day after the onset of symptoms was normal; however, it did show the abnormalities of the white substance in the fifth day.

This syndrome is usually reversible after symptomatic treatment and after suspension of the responsible agent [1, 3], but some cases have been described as nonreversible [15].

Several cytotoxic drugs are implicated in the genesis of this syndrome. An extensive search on PubMed from January 2006 to January 2014 by looking for patients who are treated by chemotherapy containing one or more of the following drugs, Bevacizumab, Oxaliplatin, and 5 Fluorouracil, and who developed encephalopathy showed that Oxaliplatin, Bevacizumab, and in a low degree 5 FU are implicated in this syndrome. Bevacizumab is a humanized monoclonal antibody that blocks VEGFA (vascular endothelial growth factor A). Its relationship with the PRLS has been reported repeatedly in the literature. Seet and collaborator have recently published two cases of PRLS secondary to Bevacizumab; among both cases, there was a patient who also received Oxaliplatin; Bevacizumab was arrested and Oxaliplatin was continued without recurrence of PRLS [17].  Table 1 shows 14 cases of leucoencephalopathy found in patients receiving chemotherapy with Bevacizumab; in most of these cases the blood pressure was high (11/14 patients) [18–29]. The Oxaliplatin is a third generation platinum known for its toxicity on the peripheral nervous system. Search on PubMed allowed for labeling five cases of PRLS due to Oxaliplatin; among these five cases, there was a patient who has received additionally capecitabine and Bevacizumab [30–34] (Table 2). 5 FU: Its relationship with PRLS has not been clearly demonstrated in the literature. It has been described as responsible of two types of toxicity on the central nervous system: acute toxicity and delayed toxicity [35]. Acute toxicity is as an encephalopathy, which can lead to coma and cerebellar syndrome. Two mechanisms have been proposed: a deficiency in the activity of dihydropyrimidine dehydrogenase [36] and hyperammonemia [37, 38]. Delayed toxicity is a multifocal inflammatory demyelinating encephalopathy [39] and the treatment is based on corticosteroids. In our case it is difficult to decide on the drug responsible for this syndrome but it appears that the combination of several drugs of chemotherapy increases the risk of occurrence of PRLS. Practitioners have to be aware of this syndrome and must recognize it early in its typical and atypical form to set up a treatment which may allow the return to the normal state.

## Figures and Tables

**Table 1:** 

Authors	Patient (age, sex)	Localization	Chemotherapy	Symptoms	MRI	Treatment	Outcomes
Sawaya et al., 2014 [18]	31 W	Ovarian carcinoma	Bevacizumab	Focal tonic-clonic seizure BP: not mentioned	Cortical and subcortical lesions of the parietal, occipital, and frontal lobes, posterior fossa, left pons, left cerebral peduncle	Intravenous benzodiazepines, phenytoin, and valproic acid	The patient recovered slowly over a fortnight

Dersch et al., 2013 [19]	41 W	Lung cancer	Gemcitabine-cisplatin Bevacizumab	Grand mal seizures, nausea, vomiting, limb ataxia, visual hallucinations, confusion, headache BP: 180/110 mmHg at admittance with peaks to 245/140 mmHg	Bifrontal, parietal, temporal, thalamic, and cerebellar laminar T2-hyperintensive lesions	—	—

Lazarus et al., 2012 [20]	72 M	Lung cancer	Maintenance Bevacizumab (patient received paclitaxel-carboplatin and Bevacizumab before)	Emesis, aphasia, altered mental status, agitation, myoclonus, tonic-clonic seizures, BP: 164/75	Bilateral cortical hyperintensities, involving the occipital lobes and cerebellar hemispheres	Enoxaparin	Worsening of lesions patient died

Lau and Paunipagar, 2011 [21]	63 W	Metastatic rectosigmoid carcinoma	Intravenous Bevacizumab in combination with oxaliplatin and 5-fluorouracil	Headache, drowsiness, visual disturbance, no focal neurological signs in the limbs vital signs were stable (BP: not mentioned)	Complete spontaneous clinical recovery within 1 week	Supportive measures	Signal abnormalities in the subcortical white matter in the posteroinferior parietotemporal lobes

Seet and Rabinstein, 2012 [17]	68 W	Metastatic non-small cell lung carcinoma	Intravenous Bevacizumab in combination with taxol and carboplatin	Headaches, confusion, nausea, and vomiting BP 221/84 mmHg	Cerebellar lesions on MRI brain	Intravenous labetatol	Neurologic recovery one day later MRI changes resolved 8 days later

Seet and Rabinstein, 2012 [17]	63 W	Advanced pancreatic carcinoma	Intravenous Bevacizumab in combination with gemcitabine and oxaliplatin	Seizures and cortical blindness BP 190/94 mmHg	Parietooccipital lesions on MRI brain	Oral antihypertensive medications	Neurologic recovery 4 days later MRI changes resolved 30 days later

Levy et al., 2009 [22]	4 M	Hepatoblastoma	Intravenous Bevacizumab in combination with gemcitabine and oxaliplatin	Seizures, headache, BP: 160/120 mmHg	Frontal and parietooccipital subcortical lesions on MRI brain	Antihypertensive medications (details not mentioned)	Neurologic deficits resolved 13 days later MRI changes resolved 21 days later

Bürki et al., 2008 [23]	33 W	Metastatic breast cancer	Intravenous Bevacizumab in combination with liposomal doxorubicin	Headaches, gastralgia, nausea, and vomiting. BP: 150/100 mmHg.	Frontal and parietooccipital subcortical lesions on MRI brain	Intravenous infusion of prednisolone, furosemide, nicardipine, and mannitol	Neurologic recovery 1 day later MRI changes resolved 4 days later

El Maalouf et al., 2008 [24]	55-year-old woman	Metastatic colon cancer	Intravenous Bevacizumab in combination with fluorouracil and leucovorin	Lethargy, dysarthria, and generalized seizures BP 190/120 mmHg	Pontomedullary lesions on MRI brain	Oral amlodipine	Neurologic deficits resolved 1 day later MRI changes resolved 21 days later

Koopman et al., 2008 [25]	49 M	Colorectal cancer	Intravenous Bevacizumab in combination with oxaliplatin and capecitabine	Unconsciousness, seizures, and urinary incontinence BP 180/100 mmHg	Occipital lesions on CT brain	Antihypertensive medications (details not mentioned)	Neurologic deficits resolved 2 days later CT brain changes resolved 6 weeks later

Peter et al., 2008 [26]	57 W	Metastatic colon carcinoma	Folfox regimen + intravenous Bevacizumab	Cortical blindness BP 140/70 mmHg	Parietooccipital subcortical lesions on MRI brain	No antihypertensive medications administered	Neurologic deficits recovered 4 weeks later MRI brain resolved 7 weeks later

Ozcan et al., 2006 [27]	52 W	Metastatic rectal adenocarcinoma	Folfox regimen + intravenous Bevacizumab	Headaches, confusion, and cortical blindness BP 172/100 mmHg	Occipital subcortical lesions on MRI brain	Antihypertensive medications (details not mentioned)	Neurologic recovery 3 days later Radiologic resolution not mentioned

Allen et al., 2006 [28]	52 M	Metastatic rectal carcinoma	FOLFIRI regimen + intravenous Bevacizumab	Headaches, bilateral cortical blindness, confusion, agitation, generalized tonic-clonic seizure. Systolic BP range 140–150 mmHg	Occipital and posterior parietal lobes subcortical lesions on MRI brain	Corticosteroids	Neurologic deficits recovered 25 days later

Glusker et al., 2006 [29]	59 W	Renal cancer	Bevacizumab	Severe lethargy. Blood pressure: 168/88 mmHg cortical blindness extensor plantar responses	Frontal and parietooccipital subcortical lesions on MRI brain	Lorazepam No medication for hypertension	Return to normal without treatment 4 days later Complete resolution of the leucoencephalopathy on MRI six weeks later

Our case	44 W	Metastatic colon cancer	Folfox + Bevacizumab	Delirium, seizures, visual disturbance, focusing signs BP: 170/90 mmHg	Frontoparietooccipital lesions on brain CT	Intravenous nicardipine Oral phenobarbital	Complete neurologic recovery

BP: blood pressure, W: women, and M: men.

**Table 2:** 

Authors	Patient (Age, sex)	Localization	Chemotherapy	Symptoms	MRI	Treatment	Outcomes
Matsunaga et al., 2012 [30]	43 W	Metastatic sigmoid cancer	Modified Folfox 6	Nausea, headache, disturbed consciousness, visual disturbance, seizures Status epilepticus hypertension	Bilateral occipital lesions on MRI	No medication for hypertension	No neurologic sequelae MRI brain resolved 40 days later

Nagata et al., 2009 [31]	35 W	Metastatic sigmoid	Folfox regimen	Convulsions, headache, and visual disturbance hypertension	Bilateral lesions on posterior lobes on MRI	Antihypertensive therapy and anticonvulsive therapy	Complete resolution of symptoms MRI changes resolved 30 days later

Sharief and Perry, 2009 [32]	59 M	Metastatic colon cancer	Folfox 6 regimen	Status epilepticus BP: 156/98	Bilateral frontal cortical lesion on MRI noncontrast CT scan of the head was normal	Seizure medications	Complete recovery in 24 h MRI brain resolved two weeks later

Pinedo et al., 2007 [33]	62 W	Metastatic adenocarcinoma of rectum	Folfox + Bevacizumab	Seizures, altered mental status, bilateral lower extremity weakness BP: 190/88	Lesions on posterior lobes on MRI	Diazepam	Complete resolution on MRI 10 days later

Skelton et al., 2007 [34]	1 F	Metastatic adenocarcinoma of rectum	Folfox	Seizures Altered mental status	Hyperintensity in the white matter of the posterior hemispheres		Resolution of lesion on repeated MRI
